# Draft Genome Sequence of Aestuariibacter halophilus Type Strain JC2043

**DOI:** 10.1128/MRA.01093-21

**Published:** 2021-12-16

**Authors:** Sarah A. Emsley, Kaysa M. Pfannmuller, Blake Ushijima, Jimmy H. Saw, Michael O. Gaylor, Patrick Videau

**Affiliations:** a Department of Biology, Southern Oregon University, Ashland, Oregon, USA; b Department of Biology and Marine Biology, University of North Carolina Wilmington, Wilmington, North Carolina, USA; c Department of Biological Sciences, The George Washington University, Washington, DC, USA; d Department of Chemistry, Dakota State University, Madison, South Dakota, USA; University of Southern California

## Abstract

Aestuariibacter halophilus strain JC2043, a Gram-negative gammaproteobacterium, is often used as a reference organism for assigning taxonomy within the family *Alteromonadaceae*. Isolates of this species have also been investigated for compound degradation (e.g., phthalates and oil) and biofilm association. Presented here is the draft genome sequence of *A. halophilus* strain JC2043.

## ANNOUNCEMENT

Aestuariibacter halophilus strain JC2043, isolated from tidal flat sediment from Ganghwa Island, South Korea, is a Gram-negative, aerobic gammaproteobacterium of the family *Alteromonadaceae* (1). First proposed by Yi et al. (1), the genus *Aestuariibacter* consists of two validly published species at the time of writing: Aestuariibacter salexigens and *A. halophilus*, with “*A. aggregatus*” (2) and “*A. litoralis*” (3) having been recently reclassified into the novel genera *Marisediminitalea* (4) and *Aliiglaciecola* (5), respectively. *Aestuariibacter* features prominently in analyses resolving the taxonomy of the *Alteromonadaceae*, with *A. halophilus* functioning as a reference taxon in the valid publication of multiple species and 10 genera (4–13). The *Alteromonadaceae* family is recognized for its biosynthetic potential, and the genus *Aestuariibacter* has been studied for its potential involvement in the degradation of various compounds [e.g., di-(2-ethylhexyl) phthalate, oil, and xylan] (14–16), symbiotic interactions in aquatic environments (17, 18), and the relationship between microbial community structure, biofilms, and water quality (19–21). In the genomic era, prokaryotic taxonomies and natural product identification rely on the public availability of whole-genome sequences (22–25). As such, the whole-genome sequence for the *A. halophilus* type strain JC2043 will bolster future analyses assessing the phylogenetic relationships, biodiversity, and metabolic potential of the *Alteromonadaceae*.

Aestuariibacter halophilus strain JC2043 (DSM 15266), acquired from Leibniz Institute DSMZ (German Collection of Microorganisms and Cell Cultures; Braunschweig, Germany), was routinely cultured on plates of glycerol artificial seawater medium solidified with 1.5% agar and incubated overnight at 35°C (26). Genomic DNA isolation was conducted on a plate culture derived from a single colony by the Microbial Genome Sequencing Center, LLC (MiGS; Pittsburgh, PA, USA), using the Qiagen DNeasy blood and tissue kit according to the manufacturer’s instructions (Hilden, Germany). Paired-end libraries (151 bp) were prepared by MiGS using the Illumina Nextera kit and run on the Illumina NextSeq 550 platform as previously described (27), producing 5,749,109 pairs of raw reads. FastQC (http://www.bioinformatics.babraham.ac.uk/projects/fastqc) was used to assess the read quality; trimming and adapter sequence removal (parameters: ktrim = r, ordered, minlen = 50, mink = 11, comp = f, k = 21, ow = t, ftm = 5, zl = 4, qtrim = rl, and trimq = 20) were performed using BBDuk within the BBMap package (http://sourceforge.net/projects/bbmap) as previously described (28). The draft genome sequence was assembled using SPAdes v. 3.14.0 using the “--careful” option and specifying kmers of 21, 33, 55, 77, 99, and 121 (29). The genome was analyzed for completeness using BUSCO v. 5.2.2 with default parameters and the bacteria_odb10 and alteromonadales_odb10 databases (30, 31).

This assembly produced 14 scaffolds with a mean coverage of 69.7× and an *N*_50_ value of 596,101 bp. The complete *A. halophilus* strain JC2043 draft genome sequence consists of 4,032,098 bp with an average G+C content of 52.90%. BUSCO scores for the genome were 99.2% and 99.9% for the bacterial (123/124 genes) and *Alteromonadales* (819/820 genes) gene sets, respectively. Genome annotation was conducted using the Prokaryotic Genome Annotation Pipeline (PGAP) (32), which annotated a total of 3,810 genes and 56 RNAs, 47 of which are tRNAs and 5 of which are rRNA sequences.

### Data availability.

This whole-genome shotgun project has been deposited at DDBJ/ENA/GenBank under accession number JAJEWP000000000.1. The version described in this paper is version JAJEWP010000000. The raw sequence reads were deposited in the SRA under accession number SRR16643417 and are associated with BioSample accession number SAMN22563708.
